# Personalized messaging enhances hospital debt collection while prosocial appeals fail: Evidence from a field experiment

**DOI:** 10.1177/20552076241277035

**Published:** 2024-10-01

**Authors:** Andris Saulitis

**Affiliations:** 1 Institute of Philosophy and Sociology, 61769 University of Latvia, Riga, Latvia; 2 Collegio Carlo Alberto, Turin, Italy

**Keywords:** Trials, behaviour change, health communications, tailoring, digital, patient credit and collection

## Abstract

**Objective:**

This study aimed to understand how different communication strategies influence patients’ behaviour in paying unpaid hospital bills. The research focused on a healthcare system where patients have to pay a significant portion of their medical costs out-of-pocket.

**Methods:**

The research was conducted in collaboration with a debt collection agency in Latvia. The field experiment involved 9196 individuals with unpaid hospital bills. These individuals received randomly assigned reminders through mobile text messages and e-mails. The study compared the effectiveness of personalized messages, which included the recipient's name, with generic reminders and messages that appealed to social norms or public good contributions.

**Results:**

The findings revealed that personalized messages, specifically those that included the recipient's name, significantly improved payment rates compared with generic reminders. Conversely, messages that used social norms or public good appeals did not have a significant impact on payment rates.

**Conclusions:**

The study highlights the importance of personalized communication strategies in improving hospital debt collection. Even simple, cost-effective modifications in communication, like adding the recipient's name, can significantly enhance payment compliance. This approach not only keep the financial books of healthcare providers balanced but also suggests that personalized strategies can be extended to other areas of healthcare management. However, while these findings are promising, they indicate that more personalized and nuanced communication strategies are needed to address the broader issue of unpaid hospital bills effectively.

## Introduction

In recent decades, healthcare systems, particularly in Europe, have increasingly adopted patient cost-sharing mechanisms.^1–9^ Cost-sharing, often encompassed within out-of-pocket (OOP) payments, refers to the financial contributions made directly by patients at the point of using health services, requiring individuals to bear a portion of their medical costs. Such mechanisms are now forming a significant part of total household expenditure in many European countries. Recent data from the Organisation for Economic Co-operation and Development (OECD) reveal that OOP payments constituted approximately 15% of health spending across EU countries, with disparities ranging from less than 10% in Luxembourg, the Netherlands and France to more than 30% in Latvia, Bulgaria, Malta and Greece.^10^ The trend of patient co-payment is part of a broader strategy to manage the financial strain on public healthcare systems, amidst ongoing debates about its effectiveness and implications for equity.^11–22^

The increasing financial burden on consumers due to these OOP costs, amidst diverse healthcare needs, is a critical factor shaping patient behaviour.^23–30^ One of the significant negative externalities of patient co-payment is the rise in hospital bad debt, which might lead to deteriorating health services. Hospital bad debt, which consists of unpaid bills left by patients, is increasingly concerning, especially in systems with high OOP costs, such as in the USA. Here, the prevalence of bad debt is substantial, with studies showing its widespread impact and association with healthcare outcomes.^31,32^ Research indicates the far-reaching impact of this bad debt, with studies like Kluender et al.^33^ finding that around 17.8% of Americans had medical debt in collections in 2020. Such financial strains often lead to aggressive collection practices, further deteriorating the wellbeing of patients.^34–37^

The growing reliance on patient cost-sharing in Europe implies that the risk of hospital bad debt could become a more pressing concern, necessitating policy attention and research focus in this area. While hospital bad debt is well-studied from an economic inequality perspective,^16,31–43^ the exploration into communication and marketing strategies for debt recovery is notably scant. Investigation into this area is critical not only for increasing hospital revenues but also for tackling broader issues like patient behaviour, healthcare accessibility and system sustainability. This is particularly true in countries like Latvia, where hospital revenues are substantially reliant on patient co-payments. Despite a cap on annual co-payment sums for a patient, set at 570 euros in 2022, a considerable portion of inpatient care bills – estimated to be in the amount of several million euros and constituting around 7% of the total co-payment amount – is not paid promptly or at all.^44–47^

The research objectives for this study are to evaluate the effectiveness of various communication strategies on patient behaviour towards unpaid public hospital bills within a healthcare system characterized by significant OOP contributions. Specifically, the study aims to examine the impact of personalized messages, particularly the inclusion of the recipient's name, on payment compliance; assess the effectiveness of moral appeals framed as social norms or public goods, considering both loss and gain perspectives; and provide insights into the broader implications of these strategies for healthcare accessibility, patient behaviour and the sustainability of health systems that rely heavily on patient contributions.

The significant reliance on patient co-payments and the low health expenditure make Latvia a relevant case for studying debt recovery strategies for hospital bills. In 2022, health expenditure in Latvia was 7.5% of GDP, compared to the European average of 10.9%.^10^ The underfunding of public healthcare system leads to long waiting times and limited access to certain treatments and medications.^48^ As a result, patients are willing to pay OOP for faster access to care or must do so for services not covered by the public system. This financial strain on patients can lead to a higher incidence of unpaid hospital bills, making debt recovery a critical issue. As the trend towards reliance on OOP becomes more evident in other countries, other healthcare systems around the world might face or already are facing the same challenges of recovering bad hospital debt. Insights from Latvia could inform the development of more effective debt recovery strategies and help ensure the financial viability of healthcare systems in similar contexts.

Behavioural insights and nudging techniques have proven effective in areas such as tax collection,^49–56^ delinquent tickets and fines^57–60^ and public utilities.^61–63^ However, utility bills, delinquent fines and tax obligations often have predictable cycles or are based on known behaviours, contrasting sharply with hospital bills, which arise from unpredictable health events. This unpredictability presents unique challenges in debt collection, differing significantly from the structured contexts of tax compliance and fines management, where individuals may have a clearer expectation of their financial responsibilities.

The study offers unique insights into a low-cost, soft-touch approach in a previously unexplored area of healthcare bill payments, providing a fresh perspective on effective debt collection without resorting to aggressive practices. Conducted in late August 2016, the randomized controlled trial with 9196 individuals was carried out in collaboration with a debt collection agency in Latvia. The experimental results underscore the importance of perceived social closeness and reciprocity in enhancing payment behaviours within public healthcare settings, demonstrating that personalization – specifically, addressing recipients by name – markedly improves payment rates. Conversely, moral appeals, framed as either social norms or contributions to public goods and irrespective of being presented as losses or gains, failed to significantly alter payment behaviours compared to a generic reminder. Moreover, the ineffectiveness of generic reminders, which showed no statistical difference from not sending a message, underscores the critical need for more personalized communication strategies in healthcare debt collection.

The paper is organized as follows. First, I outline the treatments and their theoretical background. Then, I describe the experimental design, including the setting, sample and procedure of intervention and empirical strategy for assessing the treatment effect. The remaining is analysis and discussion of the results.

## Interventions and theoretical background

This study focuses on interventions that could positively affect debt repayment behaviours by countering present-bias preferences, personalizing messages and applying social norms and public goods. It incorporates seven unique treatment messages within a factorial design, outlined in Table 1, alongside a control group that does not receive intervention. This design enables a detailed examination of each behavioural trait's impact, both in isolation and in conjunction with other traits. The remaining part of this section provides a background for each designed intervention, drawing on behavioural theory and empirical evidence from studies in the collection of various types of debt.

**Table 1.** Dimensions and assigned texts for the treatment groups in the study.

Factor		Assigned texts for the treatment groups	
Personalization	Content	Treatment line in e-mail	Mobile text message
No personalization	No content	-	-
No personalization	Simple reminder	Reminder of the hospital debt!	A reminder: you have a hospital debt, case nr. 0123456. For the solution, call:76543210
	Loss-framed public good	The unpaid bill hinders the ability of the hospital to provide services to the public!	A reminder: you have a debt to the hospital, case nr. 0123456. Unpaid bill encumbers ability for the hospital to provide services to the public! For the solution, call:76543210
	Gain-framed public good	The paid bill will increase the hospital's ability to provide services to the public!	A reminder: you owe to the hospital, case nr. 0123456. Unpaid bill encumbers ability for the hospital to provide services to the public! For the solution, call:76543210
	Social norm	Around 80% of patients pay their hospital bill on time. You are in a minority that has not done so	This is a reminder that you have a debt, case nr. 1234567. Around 80% of patients pay their hospital bill on time. You are in a minority that has not done so. Contact us to find a solution: 76543210
Debtor name	Simple reminder	[Name], reminder about the debt to the hospital!	[Name], this is a reminder that you have a debt to the hospital, case nr. 0123456. For a solution, call:76543210
	Loss-framed public good	[Name], an unpaid bill encumbers the ability of the hospital to provide services to the public!	[Name], you have a debt to the hospital, case nr. 0123456. An unpaid bill encumbers the ability of the hospital to provide services to the public! For the solution, call:76543210
	Gain-framed public good	[Name], the paid bill will increase the hospital's ability to provide services to the public!	[Name], you owe to the hospital, case nr. 0123456. Unpaid bill encumbers ability for the hospital to provide services to the public! For the solution, call:76543210

Note: Messages were sent in Latvian (see Appendices 7–9 for treatment texts in Latvian and the templates).

### Present-biased preferences: the effect of a simple reminder

Studies have shown that simple communication acts, such as sending reminders, can effectively enhance payment discipline. For example, Cadena and Schoar^64^ demonstrated that a simple text message reminder could increase timely payments. Similarly, reminders have proven effective for tax and delinquent fine collections.^50,57^ This effect can be attributed to the effect of a reminder to overcome the inclination of people to delay decisions that involve immediate costs, a phenomenon known as present-biased preferences.^65–67^ Defaulting on debt often does not have immediate consequences, leading individuals to postpone payments. However, receiving regular reminders introduces immediate costs to their wellbeing, prompting them to settle their debts.^68–70^

### Social distance: personalization of a message

The concept of social distance in debt repayment is explored by personalizing a message in terms of the debtor's name. The premise is that a lack of close ties between the lender and the borrower can increase default risks. Studies like Wilkinson-Ryan^71^ have shown that transferring debt to a third party can increase delinquency rates, while personalization can reduce social distance and improve willingness to pay.

This is evident from experimental studies on debt repayment where communicating personalized and targeted information has a potential to improve debt recovery.^72^ Haynes et al.^57^ discovered that personalized messages that mentioned the name of the addressee were more effective than generic ones in collecting delinquent fines. This effect might be attributed to the creation of reciprocal relationships, as discussed by John.^73^ Karlan et al.^74^ also found that personalized reminders, that is, including the name of a loan officer in the payment reminder, are effective for repeated borrowers, underscoring the conditional and reflexive nature of reciprocity in social interactions.

Recent studies offer mixed results on the efficacy of personalization strategies in debt collection. Uhl et al.^75^ demonstrated that personalization and targeting can stimulate customers to contact their creditor to arrange a payment. In a study involving a large energy supplier, more customers in the personalized condition agreed on a payment arrangement compared to the non-personalized condition. In contrast, Saulītis^69^ conducted three field experiments involving 32,000 borrowers, where debtors received reminders with personalized language and their first name. The results showed no significant effect, highlighting that in some contexts, particularly with defaulted consumer debts, personalized messages may be ineffective.

This study involves incorporating the debtor's name in the message, aiming to reduce the social distance that has widened due to the involvement of a third-party debt collector, distinct from the original bill issuer, the hospital. This strategy seeks to humanize the debt collection process, emphasizing reciprocal relationships where friendliness can foster cooperation rather than retaliation.^76–78^

### Social norms

Social norms, as defined by Jon Elster,^79^ encompass nonconsequentialist obligations and permissions derived from societal expectations. These norms often carry social rather than economic sanctions.^80^ Social norms and the perceived socially acceptable behaviour can significantly influence financial behaviours, as demonstrated by Trautmann and Vahu,^81^ who showed that the expectation of peers defaulting could induce similar non-payment behaviours in others. Further supporting this, various studies have noted the impact of foreclosure rates on the likelihood of strategic defaults.^82–85^

The distinction between injunctive norms (what is socially approved) and descriptive norms (what most people do) is crucial, as highlighted by Cialdini et al.^86^ Injunctive norms relate to perceptions of what behaviours are approved or disapproved by society. An example would be a message highlighting that ‘paying bills on time is the right thing to do’. Descriptive norms, on the other hand, refer to perceptions of what most people do in a given situation. For instance, a message stating that ‘most people pay their bills on time’ leverages a descriptive norm. A specific subdomain of descriptive norms is the minority descriptive norm, which highlights the behaviour of a minority group behaviour to influence the individual's behaviour. Hallsworth et al.^50^ found that a minority descriptive norm (‘Nine out of ten people in the UK pay their tax on time. You are currently in the very small minority of people who have not paid us yet’.) was more effective than a standard reminder or a plain descriptive social norm. This could be due to the emphasis on group identity, as evidenced by Wenzel^87^ in the context of tax payments.

In this study, the minority descriptive norm is communicated by informing individuals that the majority pay their bills on time, indicating that the individual belongs to the minority who does not meet their obligations. This approach positions debt repayment not just as a financial duty but as a moral action, aligning with broader societal values and reinforcing the debtor's role within a moral community that has long been one of the key communication strategies in debt recovery.^88,89^

### Public goods outcome

In the context of public goods and debt repayment, the notion of public goods outcomes is crucial. Public goods, as explained by Bicchieri,^90^ are benefits that, while possibly entailing individual costs, can lead to collective gains. Cooperation in this framework is conditional; individuals tend to cooperate only if they believe others will too. This is particularly relevant in the healthcare system of Latvia, where public hospitals play a significant role in providing healthcare services. Latvia's healthcare system relies heavily on public hospitals, which are funded through a combination of government budgets and patient co-payments.^10^ These hospitals are crucial in delivering essential medical services to the population, especially in a system where private healthcare options are limited and often expensive.^48^

This study examines the impact of public goods messages on debt repayment, framed as a loss or a gain. This framing is based on Kahneman and Tversky's^91^ prospect theory, which posits that people react differently to the perception of gains and losses. The loss-framed message emphasizes the negative impact of unpaid bills on the hospital's ability to provide services, while the gain-framed message focuses on the positive impact of paid bills. Previous studies have explored framing effects in various contexts. Ganzach and Karsahi^92^ found significant effects of loss-framed messages in credit card product offerings, but Karlan et al.^74^ did not observe such effect in loan payment reminders. This discrepancy suggests that framing effects might vary according to the context and the nature of the financial decision.

In the realm of taxation, the framing effects have been mixed. Hallsworth et al.^50^ found that both gain- and loss-framed messages increased compliance with tax payments, with no significant differences between the two. However, gender differences were observed, with men more responsive to messages framed as a loss. Similarly, Hasseldine and Hite^93^ observed that men were more influenced by messages framed as a loss, while women responded better to messages framed as a gain.

## Experimental setting and design

In Latvia, co-payment for health services at public hospitals is mandatory for nearly all patients, with specific groups such as children and the poor being exempt. At the time of the experiment, the cost ranged from €1.42 to €35.57 per service, with caps placed at €355.72 for a single treatment and €569.15 annually. Patients seeking services without a doctor's referral face no cost caps, resulting in potentially higher expenses borne entirely by the individual.

Outpatient care requires payment before service, while inpatient care, billed after treatment, may be subject to avoidance due to this post-treatment billing process. Although hospitals cannot refuse intensive or emergency care due to unpaid bills, elective services can be refused. The absence of a unified debtor list allows individuals with outstanding bills to seek elective healthcare at different hospitals, effectively reducing the immediate consequences of non-payment.

**Figure 1.**
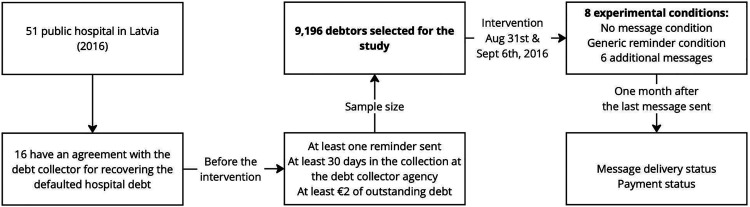
Experimental design.

In light of these healthcare policies and the challenges they present, the experimental study was carried out in 2016. In the following subsections, I will explain in detail the sample, experimental procedure and empirical method of the study. These subsections will provide a comprehensive understanding of the study's methodology, whereas Figure 1 highlights the most important points.

### The sample

At the launch of the experiment, Latvia had 51 public hospitals, with 16 of them having an agreement with the debt collection agency involved in the study. The sample offered a balanced representation in terms of hospital type and geographical distribution, with approximately one-third located in the capital, Riga, and the remainder spread across various regions. Eligibility for the experiment required individuals to meet three criteria: a single active debt collection case with the agency, a minimum debt of €2 and at least 30 days in the collection process with a prior generic payment reminder sent by the debt collection company.

As a result, the experiment involved 9196 distinct debtors. The data provided by the debt collection agency included detailed information on each case, such as the debtor's region of residence, associated hospital, first name, invoice date and duration of the debt collection process (see Appendix 1 for descriptive statistics). A significant portion (65%) of individuals were under the collection process by the debt collector for more than a year. Debt amounts ranged from €2 to €3802.55, averaging approximately €69, inclusive of collection fees. Contact information varied, with 79% having only a phone number and 20% possessing both phone and e-mail.

Based on the received data, additional variables were constructed for both the study's randomization and result analysis. These included identifying the debtor's gender, ethnicity (Latvian or non-Latvian) and age as of August 31, 2016. Furthermore, the distance between the debtor's residence at the county level and the hospital was calculated. Drawing parallels to tax collection research, evidence suggests that local expenditure of revenues might enhance payment rates compared to federal allocation, with decentralization potentially leading to increased compliance (^94–96^, Ch. 5).

### Experimental procedure

To ensure a rigorous evaluation of interventions aimed at enhancing debt repayment behaviours, the experimental procedure barred any communication from the debt collector to the debtors in the sample for 30 days prior to the experiment and until the final assessment of payment activities. Through blocked randomization, individuals were assigned to one of the eight experimental conditions, including a control group receiving no messages and seven treatment groups, each receiving a designated message. This methodological approach guaranteed that any differences observed in debtor responses could be attributed to the interventions rather than pre-existing disparities (see Appendix 1 for the variables and descriptive statistics for each experimental condition).

Both e-mails and text messages were sent at the same time via the debt collection automated system. The first round of messages was sent on Wednesday, 31 August 2016. On the following Tuesday, subjects who have not paid back the debt or signed up for a repayment plan received the same assigned message from the debt collector repeatedly. As a result, the debtor received a maximum of two identical e-mails and mobile text messages from 31 August to 6 September 2016.

In case of a missing phone number or e-mail address, the debtor received treatment through only one channel. The availability of communication channels was used during blocked randomization. As a result, individuals with only one communication channel were evenly distributed among the experimental conditions (see Appendix 2 for the proportion of communication channels used in each experimental condition).

### Statistical analysis

The variable of interest in the experiment is the payment rate, coded as ‘1’ for debtors who initiated the repayment and ‘0’ for those who did not. The debt collector prepared the report on each individual's payment activity on 4 October 2016, one month after the final intervention. Having a month between the last intervention and the check on payment activity ensured that the debtor had enough time to respond to the intervention and make a payment.

This study aims to identify the average treatment effect (ATE) by comparing payment rates across experimental conditions. The estimation was carried out by fitting a linear probability model using ordinary least squares regression, since the variable of interest is binary, with covariate adjustments due to heterogeneity within the sample.^97^ Specifically, the regression is formulated as follows:

(1) $$\begin{array}{rl} P_{i} & =\alpha +\beta_{0}M_{i}+\beta_{1}P_{i}+\beta_{3}L_{i}+\beta_{4}G_{i}+\beta_{5}S_{i}\;\;\\ & \;+\gamma_{1}(P_{i}G_{i})+\gamma_{1}(P_{i}L_{i})\;+\epsilon_{i} \end{array}$$

$P_{i}$ is a dummy variable that indicates the probability of an individual *i* to initiate the repayment within the 30 days after the first messages were sent. $M_{i}$ is a treatment dummy indicating if the individual was part of the control group that received a generic reminder or was allocated to the untreated No Message group. $P_{i},L_{i},\;G_{i}$ and $S_{i}$ are treatment dummies that denote whether an individual received a message framed as personalization, loss, gain or social norm, respectively. The model also includes interaction terms $\left(P_{i}\;X\;L_{i}\right)$ and $\left(P_{i}\;X\;G_{i}\right)$ , represented by coefficients $\gamma_{1}\;$ and $\gamma_{2}$ , to capture the combined effects of personalization with gain and loss frames.

To provide a comprehensive analysis of the treatment effects, I examine the personalization effect and the content of the messages separately. This is possible due to the factorial design of the study, which allows for the independent assessment of these factors. In the first analysis, I exclude the social norm treatment due to the lack of a comparable condition, as all messages incorporating social norms were sent in a non-personalized format. Subsequently, I categorize the remaining messages into three groups: personalized, non-personalized and No Message condition. The regression model for this analysis is specified as follows:

(2) $$P_{i}=\alpha +\beta_{0}M_{i}+\beta_{1}P_{i}\;+\epsilon_{i}$$

In this model, $M_{i}$ is a dummy variable indicating if the individual was part of the No Message condition, while $P_{i}$ represents whether the individual received a personalized message. In the second analysis, I focus on the content of the messages, disregarding the level of personalization. I merge treatments based on the content of the messages into four categories: loss-framed, gain-framed, social norm and no message. This approach allows for an assessment of the message content on repayment behaviour, independent of personalization. The regression model for this content-based analysis is formulated as follows:

(3) $$P_{i}=\alpha +\beta_{0}M_{i}+\beta_{1}L_{i}+\beta_{2}G_{i}+\beta_{3}S_{i}+\epsilon_{i}$$

In this equation, $L_{i},\;G_{i}$ and $S_{i}$ are dummy variables indicating whether the individual received a message framed around loss, gain or social norm, respectively, and $M_{1}$ remains as the dummy variable for the No Message condition. By using models described in formulas (2) and (3), I am able to isolate and analyse the effects of personalization and message content separately, providing a clearer understanding of how each factor influences repayment behaviour. The factorial design of the study ensures that the interaction between different treatment components can be systematically explored, thereby enhancing the robustness and validity of the findings.

Considering that non-compliance is common in field experiments, I focus on individuals who received the message for a more accurate assessment of the treatment effect.^98,99^ The identification of compliers was done by collecting additional data from the debt collector's communication software platform that reports on message delivery for each recipient. To calculate Complaint Average Causal Effect (CACE) for any treatment condition that was assigned to receive any message, I apply the same regression model as used to calculate ATE by simply removing non-compliers from the sample.

For the No Message condition, a two-stage least squares (2SLS) regression is used, which is equivalent to an instrumental variable regression with the delivery rate used as an instrument. In summary, the CACE for the No Message group is estimated through a two-stage least–squares regression, where the first stage predicts treatment assignment based on the delivery rate and the second stage regresses the pay rate on the predicted treatment assignment. The first stage of the 2SLS regression predicts the likelihood of an individual receiving the No Message treatment based on the instrument, which is the delivery rate:

(4) $$NoMessage_{i}=\pi_{0}+\pi_{1}DeliveryRate_{i}+\upsilon_{i}$$

In this equation, $NoMessage_{i}$ is a dummy variable indicating if individual *i* was in the No Message group, and $DeliveryRate_{i}$ is the instrument indicating the rate at which messages were delivered among the all other experimental conditions; $\upsilon_{i}$ is the error term. The second stage uses the predicted values from the first stage to estimate the effect of the No Message treatment on the pay rate:

(5) $$P_{i}=\alpha +{\hat{\beta NoMessage}}_{i}+\epsilon_{i}$$

In this equation, ${\hat{\beta NoMessage}}_{i}$ is the predicted value from the first stage regression; *β* is the coefficient representing the effect of being in the No Message group; and $\epsilon_{i}$ is the error term. By using the delivery rate as an instrument, the 2SLS regression isolates the variation in the No Message treatment that is due to random assignment, thereby providing a more accurate estimate of the causal effect. This approach allows for a more precise measurement of the treatment effect by accounting for non-compliance also in the group that does not receive a message. In what follows, I report experimental results on compliers, the so-called CACE, with the regression results on full sample available in the Appendix.

## Experimental results

**Figure 2.**
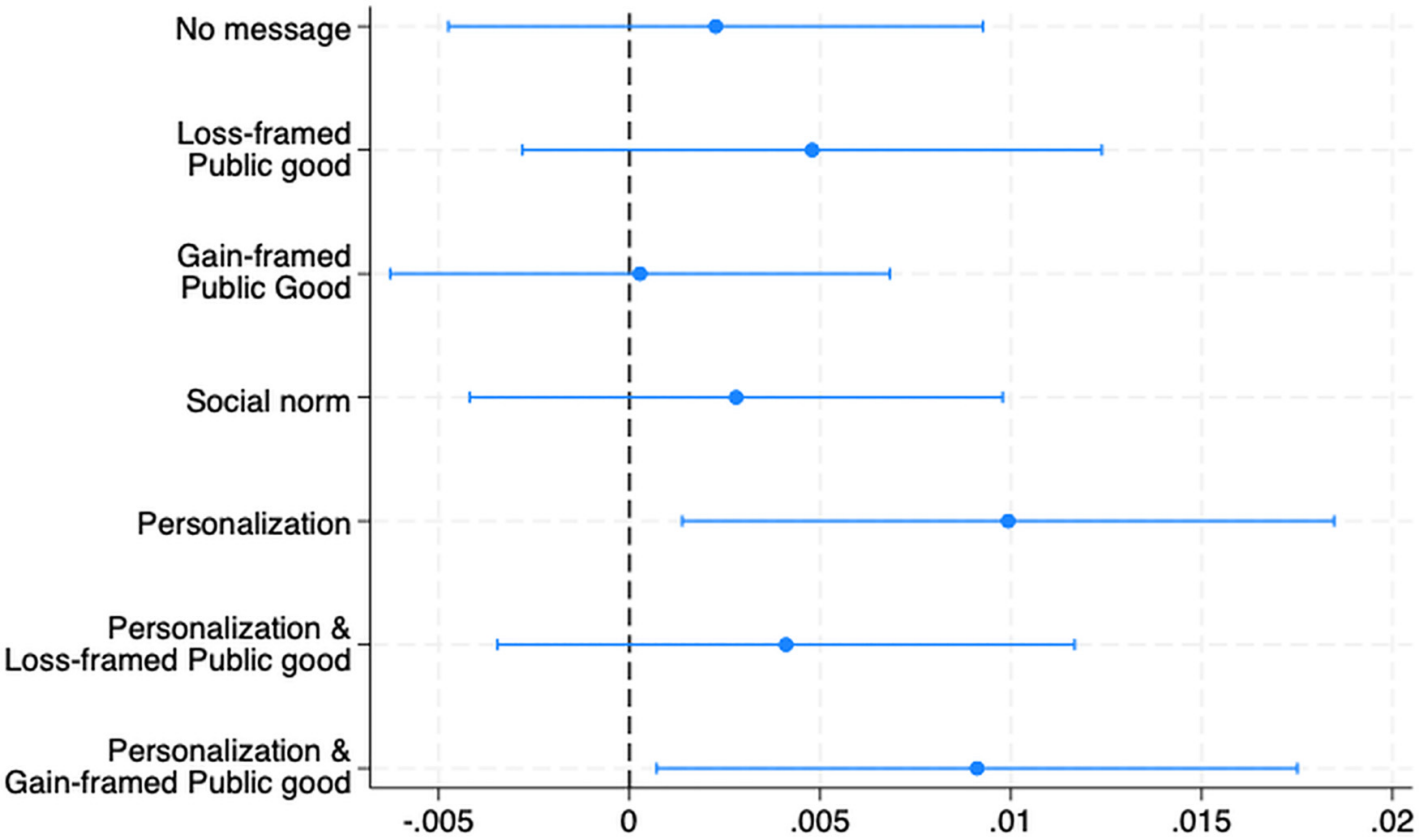
Treatment effect in average marginal effects: full sample (baseline: simple reminder).

*Note:* marginal effects from regression model with covariates (see Appendix 3, Model (4)).

The observed payment rate in the sample was remarkably low, approximately 1%, which was anticipated given the prolonged nature of the debts and previous unsuccessful recovery efforts, including communications from both the debt collector and hospitals. The payment rate varies from 0.6% to 1.7% depending on the experimental conditions (see Figure 2). The low message delivery rate (46%; see Appendix 2 for delivery rates in each condition) underscores the rationale behind concentrating the analysis on individuals who actually received the messages, which ensures the examination remains grounded in the context of the intervention's reach.

The generic reminder did not significantly improve the payment rates above the No Message condition (see Table 2). On the contrary, the introduction of personalization in the messages significantly improved the payment rate compared to other messages, with two conditions featuring the debtor's name achieving statistical significance (*p* < 0.05), after adjusting for covariates.

**Table 2.** Compliance average causal effect of text reminders on payment rate.

	(1)	(2)
Treatment (baseline: simple reminder)		
No message	0.0048	0.0046
	(0.0064)	(0.0063)
Loss-framed public good	0.0107	0.0109
	(0.0080)	(0.0080)
Gain-framed public good	0.0018	0.0022
	(0.0067)	(0.0067)
Social norm	0.0055	0.0060
	(0.0072)	(0.0071)
Personalization	0.0230*	0.0232*
	(0.0092)	(0.0091)
Personalization and loss-framed public good	0.0070	0.0057
	(0.0074)	(0.0074)
Personalization and gain-framed public good	0.0158	0.0164*
	(0.0083)	(0.0082)
Controlled for covariates	No	Yes
Constant	0.0113*	0.0817**
	(0.0046)	(0.0251)

*Notes: *Standard errors in parentheses; *p < 0.05; **p < 0.01; ***p < 0.001; CACE for No Message condition calculated using two-least two-stage least-squares regression with the average delivery rate across all other conditions used as an instrument. Full regression results are available in Appendices 3 and 4.

Further analysis of the results by grouping treatments by content reveals that neither social norms nor public goods messages framed in any manner significantly outperformed the generic reminder (see Appendix 5). Payment rates hover around 2% across this dimension. At the same time, personalizing the message produces a statistically significant increase in payment rates (*p* < 0.05; see Appendix 6) by approximately one percentage point relative to the No Personalization condition, highlighting personalization as a crucial factor in improving payment responses among compliers, with a personal touch proving to be more effective than content-based appeals or generic communication.

### Exploratory analysis

There are several control variables that correlate strongly with the payment rate (see Appendix 3). Specifically, an increase in debtor age correlates with a higher likelihood of payment; each decade of age increases the payment rate by 0.23 percentage points, a statistically significant change (*p* < 0.05). Furthermore, gender differences are apparent, with women being 0.6 percentage points more likely to repay their debts, aligning with existing research on gender disparities in tax studies.^100–102^ As men make up more than two-thirds of those included in the experiment, the findings here could reasonably be construed as describing only the behaviour of the predominant gender in the sample.^103^ However, the gender subgroup analysis of the experimental results does not show statistically significant differences between men and women under various experimental conditions.

As debt increases, repayment rates tend to decrease, highlighting a deterrent effect of larger financial obligations on debtor's willingness to pay. Furthermore, an incremental increase in collection fees relative to the total debt amount significantly impacts the probability of repayment. Specifically, a 10% increase in the ratio of collection fees to total debt decreases the likelihood of repayment by 0.24 percentage points. This effect becomes more pronounced as collection fees reach 50% of the total debt, reducing the probability of payment to practically zero compared to scenarios where collection fees are minimal or constitute a smaller fraction of the debt.

Additionally, the duration for which a bill has been in collection inversely affects the likelihood of payment, underscoring the challenge of changing behaviour over time. In contrast, the distance from the hospital does not show a significant impact on payment rates, indicating that factors related to the location of the service do not influence debt repayment behaviours. These nuanced findings offer valuable information on the dynamics that affect debt repayment, contributing to a deeper understanding of financial behaviour in the context of healthcare debt.

## Discussion

This study investigated the dynamics of hospital bill payments among debtors in Latvia, focusing on the effectiveness of various communication strategies. It aimed to discern whether different forms of messaging could influence the likelihood of debt repayment in a healthcare setting. Working with a specific type of debtors who have not paid their bills after having received at least one reminder from a debt collector, the experimental results reveal a greater understanding and opportunity for public institutions that collect co-payments for their services to use behavioural insights to improve their balance sheets.

This research significantly contributes to the literature by demonstrating the effectiveness of personalized communication strategies in the context of hospital bill collections, an area that has received limited attention. By comparing the efficacy of various messaging strategies, this study provides robust evidence that personalization, rather than generic or moral appeal-based reminders, is more effective in enhancing payment compliance. This aligns with and expands upon existing research in behavioural economics and public policy, such as the works of Hallsworth et al.,^50^ who explored social norms and public goods messages in tax compliance, and Haynes et al.,^57^ who investigated personalized messages in the context of delinquent fines.

First, the study shows the causal null effect of sending a generic reminder on hospital bill payment when the debtor has defaulted on the debt and already received at least one generic reminder in the past. This is in contrast to previous studies that have shown the positive effect of generic reminders and repeated reminders both in consumer debts and various fines from state and municipal authorities.^57,58,68,69^ The finding is more in line with the study by Migchelbrink and Raymaekers,^59^ who find that simplification does not improve the collection of parking fines. It indicates that a more nuanced approach, integrating more advanced nudges based on behavioural insights, might be required for hospital debt collections. For instance, Migchelbrink and Raymaekers^59^ found that combining several messages in one, namely, simplification, deterrence and social norms, increases the probability of paying parking fines on time. However, in this study no combination of different treatments delivers an improved payment.

One of the most surprising findings of this study is that moral appeals in the form of social norm or public good message do not improve the payment rate for public hospital debts. This is in sharp contrast to the studies in tax compliance, where receiving public good services usually results in improved compliance both in the field experiments.^95,104–107^ However, this does not imply that moral considerations are irrelevant in the context of public hospital debt collection. Together with the finding of the negative impact of collection fees on repayment rates, it suggests that perceptions of fairness of debtors play an important role in decisions with regard to the contributions towards the public good.^108–112^ Individuals who believe healthcare should be free may resist co-payment, especially if it benefits a third party like a debt collector. In this context, people may not be moved when primed to moral appeals or the notion of public good delivery.

The key discovery of this research is the positive impact of message personalization on debt collection efforts, showing a 1% point increase in payment rates when the debtor's name is included in the message. This outcome, although modest compared to the 3% point improvement found by Haynes et al.^57^ in the context of delinquent fines in the UK, is noteworthy given the much lower baseline payment rate of 1% in the current study. Despite the smaller effect size, personalization effectively doubled the response rate compared to non-personalized communications, underscoring its potential to catalyse behavioural change even among individuals with a prolonged history of non-payment.

Overall, the findings of this study have significant implications for the design and implementation of debt recovery strategies in healthcare settings. They suggest that while generic reminders and moral appeals may have limited effectiveness, personalized messages that reduce social distance and foster a sense of reciprocity can significantly improve payment rates. This highlights the need for healthcare providers and policymakers to adopt more personalized and nuanced communication strategies to address the challenge of unpaid hospital bills, ensuring the financial sustainability of healthcare systems. However, it is essential to acknowledge that while nudging and, particularly, personalized communication can improve payment compliance, they may not be sufficient to address the broader burden of bad hospital debt. Overreliance on OOP payments can have negative consequences on healthcare accessibility, disproportionately affecting low-income patients and exacerbating health inequities.^113^ Therefore, while implementing personalized strategies is a step forward, it must be complemented by broader policy reforms aimed at reducing the financial burden on patients and ensuring equitable access to healthcare services. Policymakers should consider integrating these findings with other systemic changes, such as increasing public funding for healthcare.

Beyond the primary focus of this study on patients with outstanding bills, the effectiveness of personalization points to the transformative power of customized healthcare interventions. By applying personalized strategies across the healthcare spectrum, there is a significant opportunity to improve public health outcomes, increase vaccination rates and meet individual health needs more effectively.^114–117^ This study's finding that even minimal, cost-effective interventions like personalized messaging can significantly enhance payment compliance advocates for a more individualized approach in healthcare practices. This insight not only promotes better financial outcomes for healthcare providers but also reinforces the importance of tailored communication in fostering a more responsive and patient-centred healthcare environment.

This study has several limitations that should be acknowledged. First, the field experiment was conducted exclusively within the Latvian healthcare system, which may limit the generalizability of the findings to other countries with different healthcare structures and payment models. Second, the reliance on text messages and e-mails as the primary modes of communication may not capture the effectiveness of other potential channels such as phone calls or in-person visits, which could yield different results. Additionally, the collection was done via an outsourced company, i.e., a debt servicing agency, which might undermine the effect of public good or social norm messages, as the collection is carried out by a tendered private company.

Moreover, the study focused on a specific subset of debtors who had already received at least one reminder, potentially excluding insights from those who are new to the debt collection process. On the other hand, the ability to budge at least some individuals from this specific sample of debtors who have received several reminders with a simple personalized message brings an optimistic view on nudging. Future collaborations between healthcare institutions and researchers should investigate the effect of various reminders in diverse healthcare settings, communication channels and among the samples with different debtor characteristics. Lastly, while the study highlights the immediate impact of personalized messages, it does not explore the long-term effects of such interventions on payment behaviour or patient satisfaction. Conducting longitudinal analyses would help to better understand the sustained impact of personalized debt recovery strategies and perhaps address the roots of the non-payment.

## Conclusions

Despite its limitations, this study offers significant insights into effective strategies for enhancing hospital bill payments. The findings underscore the critical role of personalized communication, revealing that messages including the recipient's name can significantly improve payment rates compared to generic reminders. Conversely, moral appeals framed as social norms or public goods did not show significant impact. While nudging through personalized communication can improve compliance, it is not a standalone solution for the broader burden of hospital debt. Personalized strategies should be complemented by policy reforms aimed at reducing financial burdens on patients and ensuring equitable access to healthcare services.

## Figures and Tables

**Appendix 7.**
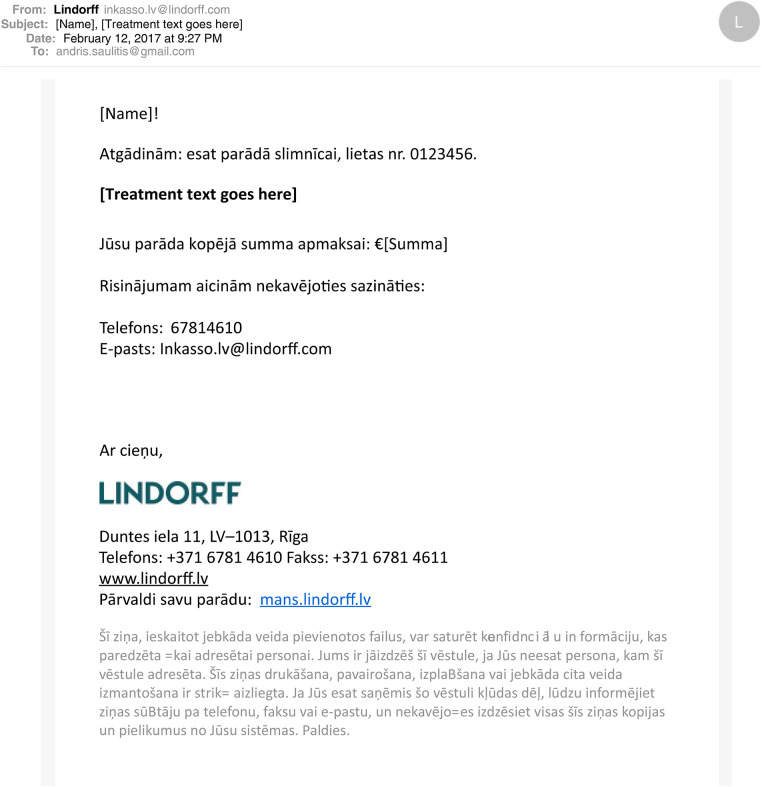
E-mail template in Latvian.

**Appendix 8.**
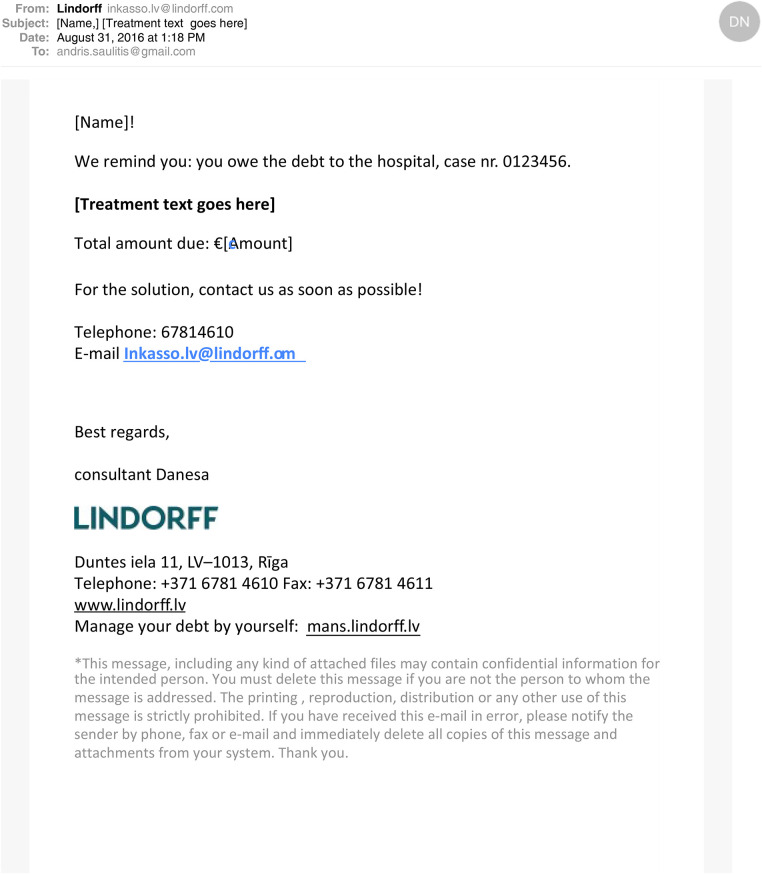
E-mail template translation in English.

## Appendix

**Appendix 1.** General statistics (means) for the control and treatment groups.

Covariate	Simple reminder	No message	Loss-framed public hood	Gain-framed public good	Social norm	Personalization	Personalization and loss-framed public good	Personalization & gain-framed public good	*p*-value from joint orthogonality test of treatment arms
Gender	0.677	0.705	0.681	0.660	0.697	0.661	0.672	0.685	0.239
	(0.014)	(0.013)	(0.014)	(0.014)	(0.014)	(0.014)	(0.014)	(0.014)	
Debt amount (log)	3.826	3.820	3.791	3.861	3.845	3.866	3.905	3.828	0.096
	(0.026)	(0.026)	(0.026)	(0.027)	(0.026)	(0.027)	(0.026)	(0.027)	
Fee ratio	0.387	0.384	0.388	0.369	0.378	0.376	0.358	0.379	0.064
	(0.007)	(0.007)	(0.007)	(0.007)	(0.007)	(0.007)	(0.007)	(0.007)	
Ethnicity	0.332	0.318	0.339	0.341	0.344	0.358	0.355	0.357	0.452
	(0.014)	(0.014)	(0.014)	(0.014)	(0.014)	(0.014)	(0.014)	(0.014)	
Debtor age	41.338	40.906	41.254	41.128	40.591	41.687	42.342	41.597	0.141
	(0.429)	(0.424)	(0.427)	(0.421)	(0.411)	(0.434)	(0.433)	(0.432)	
Debt due age	1.715	1.644	1.642	1.656	1.688	1.645	1.612	1.647	0.473
	(0.033)	(0.033)	(0.032)	(0.033)	(0.032)	(0.032)	(0.032)	(0.032)	
Distance	21.236	21.627	26.959	21.570	24.855	23.652	21.495	21.957	0.085
	(1.549)	(1.243)	(1.813)	(1.249)	(1.417)	(1.975)	(1.446)	(1.516)	
Region	2.576	2.588	2.634	2.580	2.665	2.581	2.544	2.599	0.595
	(0.042)	(0.041)	(0.043)	(0.042)	(0.043)	(0.043)	(0.042)	(0.043)	
Delivery channel	1.391	1.453	1.379	1.412	1.435	1.403	1.402	1.397	0.401
	(0.023)	(0.025)	(0.023)	(0.024)	(0.024)	(0.023)	(0.023)	(0.023)	
*N*	1150	1149	1149	1150	1150	1149	1149	1150	

Note: Standard deviation in parenthesis.

**Appendix 2.** Delivery rates across the experimental conditions.

Experimental condition	Share of delivered messages
Simple reminder	46%
No message	0%
Social norm	44%
Debtor name	46%
Agent name	46%
Debtor and agent name	46%
Debtor name and social norm	46%
Agent name and social norm	47%
Debtor and agent name and social norm	48%
Total	40%

**Appendix 3.** Four OLS regression models of treatment effect on payment rate: all conditions.

	(1)	(2)	(3)	(4)
	Treated only	Treated only	Full sample	Full sample
Treatment (baseline: simple reminder)				
No message			0.0026	0.0023
			(0.0036)	(0.0036)
Loss-framed PG	0.0107	0.0109	0.0052	0.0048
	(0.0080)	(0.0080)	(0.0039)	(0.0039)
Gain-framed PG	0.0018	0.0022	0.0009	0.0003
	(0.0067)	(0.0067)	(0.0034)	(0.0033)
Social norm	0.0055	0.0060	0.0026	0.0028
	(0.0072)	(0.0071)	(0.0036)	(0.0036)
Personalization	0.0230*	0.0232*	0.0104*	0.0099*
	(0.0092)	(0.0091)	(0.0044)	(0.0044)
Personalization and loss-framed PG	0.0070	0.0057	0.0052	0.0041
	(0.0074)	(0.0074)	(0.0039)	(0.0039)
Personalization and gain-framed PG	0.0158	0.0164*	0.0096*	0.0091*
	(0.0083)	(0.0082)	(0.0043)	(0.0043)
Gender		−0.0083		−0.0064*
		(0.0051)		(0.0025)
Debt amount (log)		−0.0118*		−0.0064**
		(0.0048)		(0.0025)
Collection fee ratio		−0.0482**		−0.0242**
		(0.0153)		(0.0078)
Ethnicity		−0.0069		−0.0036
		(0.0050)		(0.0022)
Debtor age		0.0006**		0.0002**
		(0.0002)		(0.0001)
Debt due age		−0.0098***		−0.0060***
		(0.0023)		(0.0011)
Distance to hospital		−0.0000		−0.0000
		(0.0000)		(0.0000)
Region	No	Yes	No	Yes
Treatment delivery channel	No	Yes	No	Yes
Constant	0.0113*	0.0817**	0.0061**	0.0475***
	(0.0046)	(0.0251)	(0.0023)	(0.0127)
*N*	3719	3719	9196	9196
*r*2	0.0028	0.0232	0.0012	0.0116

Standard errors in parentheses.

**p *< 0.05; ***p *< 0.01; ****p *< 0.001.

**Appendix 4.** Compliant average causal effect of communication on the payment rate (two-stage least squares).

	(1)	(2)
CACE for no message	0.0048	0.0046
	(0.0064)	(0.0063)
Gender		−0.0063*
		(0.0026)
Debt amount (log)		−0.0063**
		(0.0024)
Collection fee ratio		−0.0235**
		(0.0078)
Ethnicity		−0.0034
		(0.0022)
Debtor age		0.0002*
		(0.0001)
Debt due age		−0.0060***
		(0.0011)
Distance to hospital		−0.0000
		(0.0000)
Region	No	Yes
Treatment delivery channel	No	Yes
Constant	0.0087**	0.0497***
	(0.0027)	(0.0126)
*N*	9196	9196
*r*2	0.0031	0.0131

Standard errors in parentheses.

**p *< 0.05; ***p *< 0.01; ****p *< 0.001.

**Appendix 5.** Four OLS regression models for content of the message effect on payment rate.

	(1)	(2)	(3)	(4)
	Treated only	Treated only	Full sample	Full sample
Treatment (baseline: simple reminder)				
No message			−0.0026	−0.0027
			(0.0035)	(0.0035)
Gain-framed PG	−0.0026	−0.0021	−0.0000	−0.0003
	(0.0063)	(0.0062)	(0.0031)	(0.0031)
Loss-framed PG	−0.0027	−0.0034	0.0000	−0.0005
	(0.0063)	(0.0063)	(0.0031)	(0.0031)
Social norm	−0.0059	−0.0055	−0.0026	−0.0022
	(0.0072)	(0.0071)	(0.0035)	(0.0035)
Gender		−0.0084		−0.0064*
		(0.0051)		(0.0025)
Debt amount (log)		−0.0119*		−0.0064**
		(0.0048)		(0.0025)
Collection fee ratio		−0.0477**		−0.0241**
		(0.0153)		(0.0078)
Ethnicity		−0.0068		−0.0035
		(0.0050)		(0.0022)
Debtor age		0.0006**		0.0002**
		(0.0002)		(0.0001)
Debt due age		−0.0099***		−0.0061***
		(0.0024)		(0.0011)
Distance to hospital		−0.0000		−0.0000
		(0.0000)		(0.0000)
Region	No	Yes	No	Yes
				
Treatment delivery channel	No	Yes	No	Yes
				
Constant	0.0228***	0.0935***	0.0113***	0.0523***
	(0.0046)	(0.0253)	(0.0022)	(0.0127)
*N*	3719	3719	9196	9196
*R* ^2^	0.0002	0.0205	0.0001	0.0105

Standard errors in parentheses.

**p *< 0.05; ***p *< 0.01; *** *p *< 0.001.

**Appendix 6.** Four OLS regression models for content of the message effect on payment rate.

	(1)	(2)	(3)	(4)
	Treated only	Treated only	Full sample	Full sample
Treatment (baseline: no personalization)				
No message			0.0006	0.0006
			(0.0031)	(0.0032)
Personalization	0.0112*	0.0106*	0.0064*	0.0059*
	(0.0051)	(0.0050)	(0.0025)	(0.0025)
Gender		−0.0083		−0.0067*
		(0.0056)		(0.0027)
Debt amount (log)		−0.0126*		−0.0068*
		(0.0055)		(0.0027)
Collection fee ratio		−0.0587***		−0.0283**
		(0.0172)		(0.0086)
Ethnicity		−0.0050		−0.0027
		(0.0057)		(0.0024)
Debtor age		0.0007**		0.0003**
		(0.0002)		(0.0001)
Debt due age		−0.0101***		−0.0061***
		(0.0026)		(0.0012)
Distance to hospital		−0.0000		−0.0000
		(0.0000)		(0.0000)
Region	No	Yes	No	Yes
				
Treatment delivery channel	No	Yes	No	Yes
				
Constant	0.0154***	0.0891**	0.0081***	0.0509***
	(0.0031)	(0.0273)	(0.0015)	(0.0134)
*N*	3185	3185	8046	8046
*R* ^2^	0.0015	0.0267	0.0009	0.0131

Standard errors in parentheses.

**p *< 0.05; ** *p *< 0.01; ****p *< 0.001.

**Appendix 9.** Treatment texts in Latvian.

Personalization	Group	E-mail (subject line)	Text message
No message	Control	-	-
No personalization	Simple reminder	Atgādinājums par parādu slimnīcai!	Atgadinam:esat parada slimnicai, lietas nr.0123456! Risinajumam zvani:T.67814610
	Loss-framed public good	Neapmaksātais rēķins apgrūtina iespējas slimnīcai sniegt iedzivotājiem pakalpojumus!	Atgadinam:esat parada slimnicai, lietas nr.01234567,tas apgrutina iespejas slimnicai sniegt iedzivotajiem pakalpojumus!Risinajumam zvani:T.67814610
	Gain-framed public good	Apmaksāts rēķins paplašinās iespējas slimnīcai sniegt iedzīvotājiem pakalpojumus!	Atgadinam:esat parada slimnicai,lieta nr.0123456.Apmaksats rekins paplasinas iespejas slimnicai sniegt iedzivotajiem pakalpojumus!Risinajumam zvani:67814610
	Social norm	9 no 10 pacientiem rēķinu samaksā. Esat retais, kurš nemaksā.	Atgadinam:esat parada slimnicai, lietas nr.[lietas_nr]!9 no 10 pacientiem rekinus samaksa,esat retais,kurs nemaksa.Risinajumam zvani:T.67814610
Debtor name	Simple reminder	[Name], atgādinām par parādu slimnīcai!	[Name],atgadinam:esat parada slimnicai (nr.0123456)!Risinajumam zvani:T.67814610
	Loss-framed public good	[Name], neapmaksātais rēķins apgrūtina iespējas iedzīvotājiem sniegt pakalpojumus!	[Name],esat parada slimnicai, lietas nr.0123456,tas apgrutina iespejas slimnicai sniegt iedzivotajiem pakalpojumus!Risinajumam zvani:67814610
	Gain-framed public good	[Name], apmaksāts rēķins paplašinās iespējas slimnīcai sniegt iedzīvotājiem pakalpojumus!	[nameabc],esat parada slimnicai,lieta nr.0123456.Apmaksats rekins paplasinas iespejas slimnicai sniegt iedzivotajiem pakalpojumus!Risinajumam zvani:67814610
